# Theoretical impact of a bedside decision-making tool on antibiotic use for suspected neonatal healthcare-associated infection: an observational study

**DOI:** 10.1186/s12887-024-05323-8

**Published:** 2025-01-21

**Authors:** Lizel Georgi Lloyd, Mirjam Maria van Weissenbruch, Adrie Bekker, Cecilia Ferreyra, Birgitta Gleeson, Angela Dramowski

**Affiliations:** 1 Department of Pediatrics and Child Health, Faculty of Medicine and Health Sciences, Stellenbosch University, Cape Town, South Africa; 2 Division IC Neonatology (NICU), Department of Pediatrics, VU University Medical Center, Amsterdam, Netherlands; 3 Foundation for Innovative New Diagnostics, Geneva, Switzerland

**Keywords:** Neonatal healthcare-associated infections (HAI), Neonatal sepsis, Low-and-middle incoome countries (LMICs), Antibiotic resistance, Diagnostic markers, Antibiotic stewardship

## Abstract

**Background and objectives:**

Healthcare-associated infections (HAI) are a leading contributor to morbidity and mortality in hospitalised neonates. Diagnosing neonatal HAI is challenging owing to non-specific symptoms and lack of definitive diagnostic markers, contributing to high rates of inappropriate antibiotic use. This study evaluated the theoretical impact of implementing a bedside tool for decision-making on antibiotic length of therapy (LOT).

**Methods:**

This prospective observational physician-blinded study consecutively enrolled patients with suspected HAI events at a large South African neonatal unit from September 2022 to September 2023. The antibiotic decision-making tool included an infection prediction score (NeoHoP), and a point-of-care C-reactive protein test (CRP) performed at HAI diagnosis and 24 h later. The theoretical impact of the tool on antibiotic LOT was calculated.

**Results:**

We recruited 180 neonates with 214 episodes of suspected HAI, of which 22 (10.3%) were proven HAI, 56 (26.2%) were presumed HAI and 136 (63.6%) had HAI ruled out. The median observed antibiotic LOT was three days (9 days for proven HAI, 7 days for presumed HAI, and 3 days for no HAI). The antibiotic decision-making tool would theoretically reduce overall antibiotic LOT by 2 days (*p* < 0.001), particularly in neonates where HAI was subsequently excluded.

**Conclusion:**

We developed an antibiotic decision-making tool to support the clinical evaluation of suspected neonatal HAI and demonstrated a significant potential impact on reducing antibiotic LOT. Given increasing antibiotic resistance rates globally, this tool should be further evaluated to minimise unnecessary antibiotic use in hospitalised neonates.

**Supplementary Information:**

The online version contains supplementary material available at 10.1186/s12887-024-05323-8.

## Introduction

Neonatal healthcare-associated infections (HAIs) are a severe problem in neonatal units worldwide with high morbidity and mortality, especially in very low birth weight (VLBW; <1 500 g) and premature neonates. Neonatal sepsis, an umbrella term that includes serious neonatal infections, ranks as the 3rd leading cause of neonatal mortality and constitutes 22% of overall neonatal mortality, the majority of which occurs in low-and-middle-income countries (LMIC) [1–4]. The incidence of neonatal sepsis in LMICs has been up to 40 times higher than in high-income countries, with observed mortality rates in some LMICs more than 70% [5–7].

HAIs are generally defined as infections occurring after 72 h of admission to the neonatal unit and present healthcare workers with a diagnostic challenge. Not only do neonates with HAI have non-specific clinical signs and symptoms, such as temperature instability, irritability, and feeding intolerance but there is also no consensus definition for neonatal sepsis and no ideal biomarker to identify HAI [8–11]. Consequently, antibiotic agents are the most frequently prescribed medications in neonatal units, as early appropriate treatment of neonatal infections is critical for newborn survival [12, 13]. In the NO-More-AntibioticS and Resistance (NO-MAS-R) study, a point prevalence survey in 29 countries, 26% of high-risk neonates received empiric antibiotic therapy, 83% of which received it for suspected HAI [14]. The prescription of antibiotic treatment was more common in LMICs, and the majority of these prescriptions were for ‘rule-out sepsis’ and ‘culture-negative sepsis’ [14]. Empiric antibiotic use is not without consequences for the patient, family, hospital, and broader community. The consequences include drug toxicity, unnecessary blood sampling and venous cannulation, changes in the neonatal microbiome, risk of necrotising enterocolitis and death, bronchopulmonary dysplasia, adverse neurodevelopmental outcomes, separation of the neonate from the mother, prolonged hospitalisation, increased healthcare costs, and the emergence of resistant organisms [15–18].

High use of antibiotics occurs in LMIC due to the absence or limited availability of blood culture and other laboratory diagnostics [18, 19]. Blood culture is the gold standard for sepsis diagnosis, even though this may be negative in more than half of the cases where skilled clinicians are confident of the diagnosis [3, 20]. The sensitivity of blood culture is sub-optimal in neonates because of low colony bacteraemia, limited blood volume available, and inappropriate sampling. Of note, a negative result does not exclude sepsis despite the presence of suggestive clinical signs and symptoms in the neonate [21–23].

The NO-MAS-R study assessed the length of antibiotic therapy in these neonates. The antibiotic length of therapy (LOT) was 12 days for proven infection and 7 days for presumed diseases and did not differ between high and low-middle-income countries [14]. Although the optimal antibiotic LOT is still unclear, single-centre studies have suggested that shorter antibiotic courses of 5 days are safe and effective for culture-negative infections in neonates [24–26].

Given these challenges in treating neonatal infections, the United States Centers for Disease Control and Prevention (CDC) has urged hospitals to develop systems to monitor and reduce inappropriate antibiotic use [27, 28]. In this study, we aim to develop an antibiotic decision-making tool for HAI that incorporates a combination of clinical signs and an easy-to-use diagnostic bedside test to assist healthcare workers in making informed antibiotic prescription decisions. Additionally, we will determine whether this tool has the potential to significantly reduce antibiotic LOT in this vulnerable population by two days.

## Materials and methods

### Study design and setting

This prospective observational physician-blinded study was performed at Tygerberg Hospital, a 1384-bed tertiary hospital in Cape Town, South Africa, from September 2022 to September 2023. The hospital’s busy obstetric-neonatal service manages approximately 8000 high-risk deliveries (37% low birth weight; <2500 g) and 2500–3000 neonatal admissions annually, of which 800–1000 are very low birthweight (VLBW; <1500 g) infants [29]. The neonatal unit has 132 beds, including a 12-bed Neonatal Intensive Care Unit (NICU), three high-dependency wards, and one kangaroo mother care ward.

### Patients and data collection

All neonates admitted for more than 72 h, had not received antibiotic therapy in the preceding 48 h, and were investigated for a suspected healthcare-associated infection (HAI) were eligible. Consecutive sampling was used, enrolling every neonate that met the inclusion criteria until the calculated sample size of > 126 neonates was achieved (80% power, alpha 0.05). The power calculation was based on the primary outcome, aiming to reduce antibiotic LOT by two days. Exclusion criteria were inability to obtain informed consent timeously, neonates with congenital anomalies requiring surgery, with lethal chromosomal disorders, those who had surgery in the preceding 48 h, and those with surgical necrotising enterocolitis (Bell’s stage 2b or more) [30]. Each eligible neonate was included irrespective of whether antibiotics were prescribed following laboratory work-up for suspected HAI.

### Investigation and management of neonatal HAI

Physicians in the neonatal unit decided whether suspected HAI was present, and an investigation was needed. Symptoms and signs that prompted investigation for suspected HAI included vomiting, abdominal distention, tachypnea, tachycardia, temperature and glucose instability, and mottled skin. In such cases, the attending physician followed protocol and aseptically collected a single blood culture (BC), a complete blood count (CBC), and a laboratory CRP test, with additional specimens collected for microbiological culture as clinically indicated. In almost all cases, empiric antibiotic therapy was commenced using institutional guidelines while the results of the laboratory investigations were awaiting.

### Study definitions

HAI episodes were defined as follows:

1. *Proven HAI*: a blood culture isolating a known pathogen collected after 72 h of admission associated with clinical symptoms or signs of infection. Organisms were classified using the US CDC control list of pathogens and contaminants [31]. Participants with isolated coagulase-negative staphylococci (CoNS) on blood culture/s were reviewed to distinguish between infection and contamination. CoNS infection was defined as (i) two positive blood cultures taken 24–48 h apart or (ii) a single positive blood culture combined with a serum CRP ≥ 10 mg/L and clinical features suggestive of infection (adapted from Stoll et al.) [32];
2. *Presumed HAI*: clinical symptoms of infection in the presence of a CRP ≥ 10 mg/L with a negative blood culture, where antimicrobial treatment was continued for ≥ 5 days; and
3. *No HAI*: patients with short-lived symptoms and no objective findings of infection, a negative blood culture, and a CRP < 10 mg/L, where antibiotics were discontinued within 48–72 h. The collective term of any HAI refers to proven HAI and presumed HAI.

The time to laboratory CRP result was calculated from when the blood specimen was collected from the neonate to when results were available on the National Health Laboratory Service TrakCare web viewer. Antibiotic LOT is a preferred metric for documenting antibiotic use in children because it is independent of age—and weight-related differences in dosage [33]. Antibiotic LOT was defined as the number of days a patient receives systemic antibiotic agents, irrespective of the number of antibiotics [34].

### NeoHoP score

The NeoHoP score is a novel, simple infection prediction score developed to evaluate neonates with suspected HAI episodes using five variables: capillary refill time > 3 s, lethargy, abdominal distention, presence of an in-situ central venous catheter or in the preceding 48 h, and CRP ≥ 10 mg/L [35]. Each variable is assigned a value of 1, up to a total of 5. A score of ≥ 2 is well positioned to be used as a ‘rule-in’ test, both on internal validation and in a multicenter external validation [35, 36]. For this study, we replaced the laboratory CRP test with a POC CRP test to enable rapid bedside decision-making.

### C-reactive protein

POC CRP is well established as a quick and reliable alternative to laboratory testing in resource-limited settings [37, 38]. The POC CRP for this study was conducted using the QuikRead go^®^ easy CRP from Aidian. This is an immunoturbidimetric assay for quantitatively determining CRP values in whole blood, serum, and plasma, with a measuring range of 1.0-200 mg/L on whole blood that yields a result in 2 min using 0.01 ml of blood [39]. Control testing was performed weekly per the manufacturer’s recommendations. The National Health Laboratory Service (NHLS) uses a Roche Cobas 6000 automated analyser for CRP testing. The method called latex-enhanced immunoturbidimetry used by the Roche Cobas^®^ 6000 for the CRP test is a way to measure the amount of CRP in a blood sample by mixing small particles of latex with special antibodies that recognise and stick to CRP molecules [40].

### Study procedures

A single study investigator conducted a standardised clinical assessment of the neonate at enrolment. At the time of routine laboratory specimen collection (BC, CBC, CRP), an additional whole blood sample (0.01 mL) was collected for performance of a point-of-care (POC) CRP using the Aidian QuikRead go^®^ easy CRP (designed as time point ‘t_0_’; CRP t_0h_ ), with the NeoHoP score (NeoHoP t_0h_), as described earlier [35], calculated simultaneously by the single study investigator. Urinalysis and chest X-rays were performed at the discretion of the treating clinicians when indicated, based on the clinical presentation of the neonate. Antibiotics were started after the collection of blood specimens based on the institutional guidelines as this was a physician-blinded study, and the study investigator did not interfere with the standard of care.

A second blood specimen (0.01 mL) was collected the following day, i.e., 24 h after t_0h_, via a heel prick for a second POC CRP (designed as time point ‘t_24h_’; CRP t_24h_). The time of CRP t_0h_ and the time to result in receipt for the corresponding laboratory CRP was recorded. Antibiotic LOT from the time of enrolment was documented (observed LOT). All neonates were followed up for 28 days post-enrolment or until hospital discharge or transfer out.

During the study period, the treating physician was blinded to the NeoHoP score and the subsequent POC CRP test results to ensure that any study findings did not influence antibiotic decision-making.

### Statistical analysis and development of the antibiotic decision-making tool

All statistical analysis was performed using IBM SPSS Statistics for MacIntosh, V.29.0. Means and standard deviations (SD) were calculated for normally distributed continuous variables. Medians and interquartile range (IQR) were used for non-normally distributed continuous data. For normally distributed data, independent t-test were used to compare unrelated groups; for non-normally distributed data, the Mann-Whitney U test was used. A decision tree analysis included categorical variables related to the NeoHoP score and POC CRP. We used the exhaustive Chi-squared Automatic Interaction Detection (CHAID) method to develop the decision tree [41].

The diagnosis of any HAI (i.e., proven or presumed) was used as the reference standard for correctly determining disease status in all cases. Based on this, ‘true positive’ denotes a neonate where the test and score correctly identified the presence of any HAI. ‘True negative’ indicates a neonate where the test and score correctly identified the absence of any HAI. ‘False positive’ refers to neonates where the test and score falsely identified the presence of any HAI when the neonate did not have any HAI. ‘False negative’ indicates neonates where the test and score failed to determine the presence of any HAI.

The outcome of the decision tree was then used to develop the antibiotic decision-making tool: a neonate with a NeoHoP t_0h_ of 0 requires no antibiotics and no further investigations; a NeoHoP t_0h_ of 1 requires initiation of antibiotics after appropriate laboratory investigations, followed by a CRP t_24h_ to decide on cessation or continuation of antibiotics; a NeoHoP t_0h_ of ≥ 2 at prompts a full course of antibiotics as per institutional guidelines.

Various scenarios were considered to determine the theoretical impact of the decision-making tool on antibiotic LOT. For neonates where the tool suggested non-initiation of antibiotics, the theoretical LOT was assigned as 0 days; for early cessation, the theoretical antibiotic LOT was assigned as one day; and for those where a full treatment course was suggested by the tool, the observed antibiotic LOT was used for comparative analyses.

The manuscript was prepared according to the STROBE-NI criteria [42]. The Stellenbosch University Health Research Ethics Committee and the Tygerberg Hospital management reviewed and approved the study protocol (S20/11/325).

## Results

### Description of the study population

Over a 1-year period, 180 neonates were enrolled with 214 suspected HAI episodes. The median birth weight and gestational age were 1088 g and 29 weeks, respectively (Table 1). Most infants were delivered via caesarean section, and 13.9% were born outside of the tertiary centre. Of the 214 clinically suspected HAI episodes, 36.4% (78/214) were diagnosed with any HAI (10.3% [22/214] proven HAI and 26.2% [56/214] had presumed HAI) (Table 2). There was a Gram-positive pathogen predominance in neonates with proven HAI (Supplementary Table 1).

**Table 1:** Baseline characteristics of the study population investigated for clinically suspected healthcare-associated infection (*n* = 180)

Variable		
**Birth weight (g)**,** median (IQR)**	1088	(900–1331)
≤ 1000 g, *n* (%)	73	(40.6)
1001–1500 g, *n* (%)	75	(41.7)
1501–2500 g, *n* (%)	18	(10.0)
> 2500 g, *n* (%)	15	(7.8)
**Gestational age at birth (weeks)**,** median (IQR)**	29	(27–31)
< 28 weeks, *n* (%)	88	(48.6)
28–31 weeks, *n* (%)	51	(28.3)
32–36 weeks, *n* (%)	24	(13.3)
≥ 37 weeks, *n* (%)	17	(9.4)
**Male sex**, *n*** (%)**	85	(47.2)
**Delivered by ceasarean section**, *n*** (%)**	110	(61.1)
**Born outside of tertiary facility**, *n***(%)**	25	(13.9)
**Born to a mother living with HIV**, *n*** (%)**	26	(14.4)
**Any antenatal care attended**,*n*** (%)**	170	(94.4)
**Any antenatal steroids received if GA < 34 weeks**, *n*** (%)**	129/157	(82.2)
**Died**, *n*** (%)**	15	(8.3)
**Died during antibiotic therapy**, *n*** (%)**	7	(3.9)

*IQR indicates interquartile range; HIV*,* human immunodeficiency virus*

**Table 2:** Description of clinically suspected healthcare-associated infection episodes investigated during the study period (*n* = 214)

**Age at investigation**	**Median (IQR)**	
**Postnatal age at HAI investigation (days)**	16	(8–30)
Postnatal age at HAI investigation of first episode (days)	15	(7–24)
**Postmenstrual age at HAI investigation (weeks)**	32	(30–34)
Postmenstrual age at HAI investigation of first episode (weeks)	31	(30–34)
**Time to t**_**0h**_ **laboratory CRP result (hours: minutes)**	6:17	(5:00–8:31)
**Type of infection**	**n**	**%**
**No HAI**	136	(63.6)
**Any HAI**	78	(36.4)
Proven HAI	22	(10.3)
Presumed HAI	56	(26.2)
**Antibiotic treatment**	**Median**	**(IQR)**
**Observed antibiotic LOT (days)**	3	(3–7)
**No HAI**	3	(2.5-3)
**Any HAI**	7	(5–10)
Proven HAI	9	(7–14)
Presumed HAI	7	(5–9)
**Outcome 28 days after investigation for HAI**	**n**	**%**
**Still admitted in neonatal unit**	118	(55.1)
**Transferred to a step-down facility**	35	(16.4)
**Discharged home**	43	(20.1)
**Died**	18	(8.4)

*CRP indicates C-reactive protein; HAI*,* healthcare-associated infection; LOT*,* length of treatment; IQR*,* interquartile range*

Overall, the observed antibiotic LOT for any HAI as per institutional guidelines was 3 days (median; IQR, 3–7); however, eight neonates did not receive any antibiotics based on clinician discretion; neonates with proven HAI received an observed antibiotic LOT of 9 days (median; IQR, 9–14) whereas neonates with presumed HAI received an antibiotic LOT of 7 days (median; IQR, 5–10) (Table 2). The POC CRP t_0h_ was available at 2 min, whereas the corresponding laboratory CRP was available after a median of 6 h and 17 min. The crude mortality rate was 8.3% (15/180), with 46.7% (7/15) of these deaths occurring during the course of antibiotic treatment for the current HAI.

### Decision tree analysis for investigation of suspected neonatal HAI

The use of a single POC CRP test at the time of presentation (CRP t_0h_) generated 19/214 (8.9%) false negative tests and no false positive tests for the diagnosis of any HAI (Table 3). A single POC CRP t_24h_ resulted in no false positives and 9/205 (4.4%) false negatives for diagnosing any HAI. Combining POC CRP t_0h_ and t_24h_ resulted in no false positives and 2/209 (1.0%) false negative diagnoses of any HAI. The NeoHoP t_0h_ resulted in 3/214 (1.4%) false positives and 8/214 (3.7%) false negatives.

Using a POC CRP cut-off of ≥ 10 mg/L, the decision tree analysis identified a combination of two variables: NeoHoP t_0h_ and POC CRP t_24h_ (Supplementary Fig. 1), with an average predictive value of 98.1% (area under ROC curve 0.997 (95% CI 0.991–1.002). This decision tree combination performed similarly to the NeoHoP t_0h_ alone, achieving the same specificity (97.8%), a lower negative likelihood ratio (NLR) (0.10 vs. 0.010), and a higher sensitivity (89.7% vs. 98.7%) and accuracy (94.9% vs. 98.3%) (Table 3).

**Table 3:** Diagnostic accuracy of selected combined tests for the diagnosis of suspected healthcare-associated infection

	CRP* t_0h_		NeoHoP^#^ t_0h_		CRP* t_24h_		CRP* t_0h_ and t_24h_ combined		NeoHoP^#^ t_0h_ and CRP* t_24h_ combined**	
*n*	214		214		205		209		214	
**True positive**, *n*** (%)**	60	(28.0)	70	(32.7)	65	(31.7)	76	(36.4)	77	(36.0)
**True negative**, *n*** (%)**	135	(63.1)	133	(62.1)	131	(63.9)	131	(62.7)	133	(62.1)
**False positive**, *n*** (%)**	0	(0.0)	3	(1.4)	0	(0.0)	0	(0.0)	3	(1.4)
**False negative**, *n*** (%)**	19	(8.9)	8	(3.7)	9	(4.4)	2	(1.0)	1	(0.5)
**Sensitivity (%)**	76.0		89.7		87.8		97.4		98.7	
**Specificity (%)**	100		97.8		100		100		97.8	
**Negative likelihood ratio**	0.24		0.10		0.12		0.03		0.01	
**Positive Predictive Value (%)**	100		95.9		100		100		96.3	
**Negative Predictive Value (%)**	87.7		94.3		93.6		98.5		99.3	
**Accuracy (%)**	91.1		94.9		95.6		99.0		98.1	

*CRP ≥ 10 mg/L

^#^ NeoHoP score ≥ 2

**Based on the decision tree analysis

*CRP indicates C-reactive protein; t*_*0h*_, *at the time of presentation of suspected healthcare-associated infection; NeoHoP score*,* Neonatal Healthcare-associated infection Prediction score; t*_*24h*_, *day after presentation of suspected healthcare-associated infection*

### Theoretical impact of the decision-making tool on antibiotic use

**Fig. 1:**
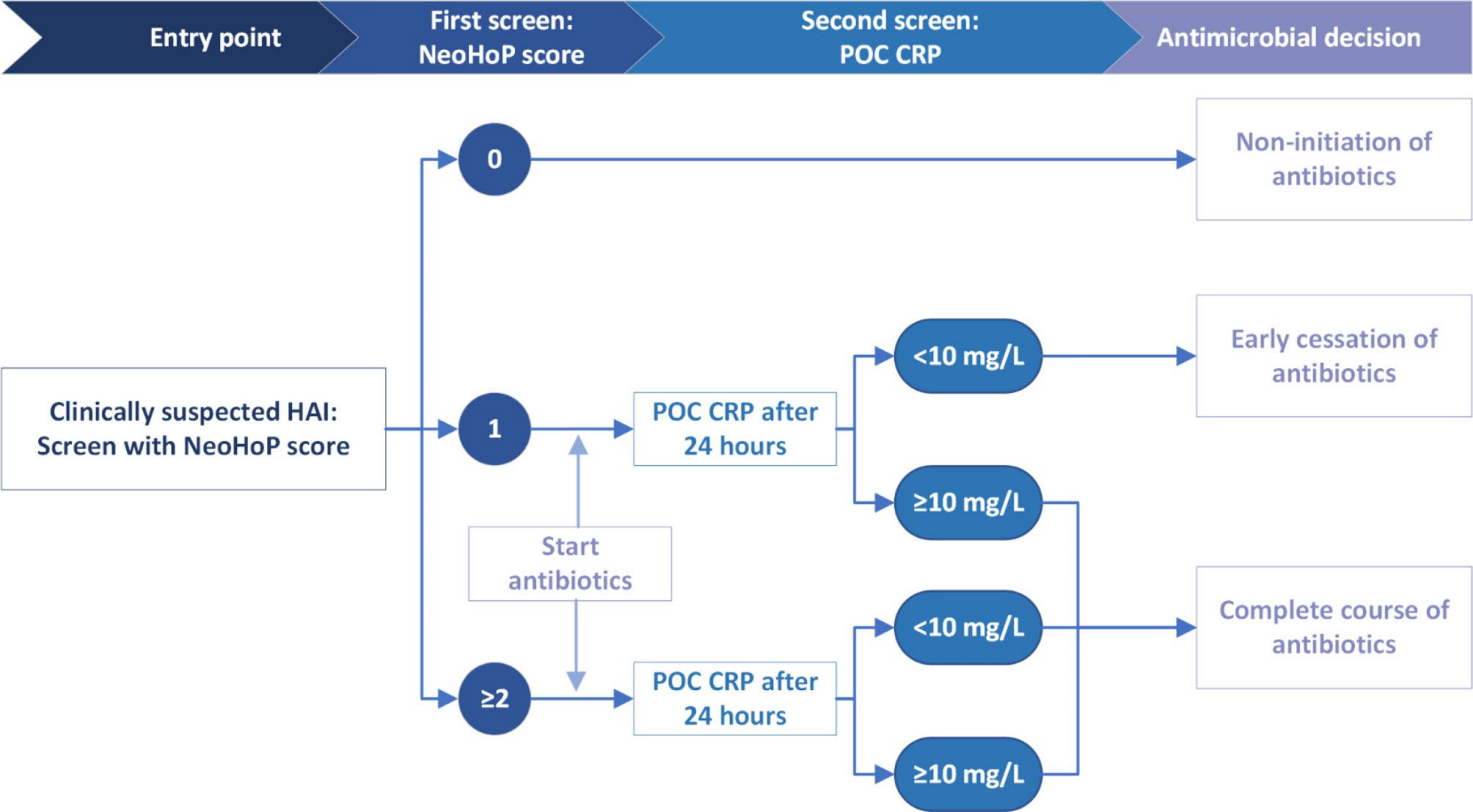
Antibiotic decision-making tool to guide antibiotic decision-making in neonates with suspected healthcare-associated infection. *HAI indicates healthcare-associated infection; NeoHoP score*,* Neonatal Healthcare-associated infection Prediction score; POC CRP*,* point-of-care C-reactive protein*

**Fig. 2:**
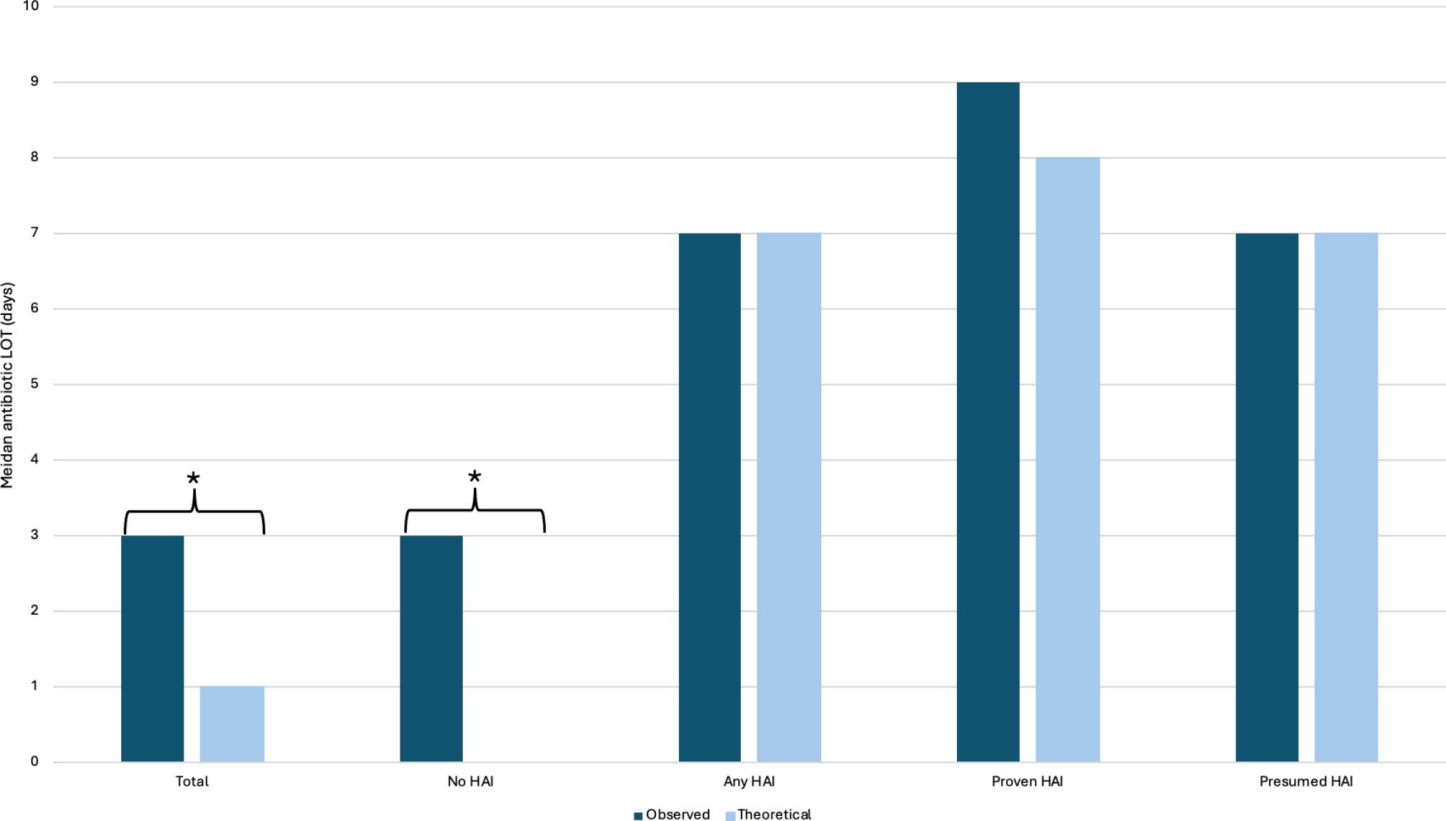
Theoretical impact of the antibiotic decision-making tool on antibiotic LOT in neonates with suspected healthcare-associated infection, compared to observed antibiotic LOT. * *p* < 0.001. *HAI indicates healthcare-associated infection; LOT*,* length of treatment*

An antibiotic decision-making tool was derived from the decision tree analysis, with a NeoHoP score (POC CRP test at t_0h_ included) followed by a POC CRP test at t_24h_. (Fig. 1). The decision-making tool was then retrospectively applied to the dataset to determine the theoretical antibiotic LOT. The decision-making tool on our data set theoretically reduced the overall antibiotic LOT for the treatment of any HAI by two days, from 3 days (median; IQR, 3–7) observed antibiotic LOT to one day (median; IQR, 0–5) theoretical antibiotic LOT (*p* < 0.001). The most substantial reduction in antibiotic LOT would be affected in the group of neonates where HAI was excluded, from 3 days (median; IQR, 2.5-3) to 0 days (median; IQR, 0–5) (*p* < 0.001) (Fig. 2, Supplementary Table 2). The decision-making tool would not affect antibiotic LOT in patients where any HAI was diagnosed, with no difference between observed and theoretical antibiotic LOT (p 1.000).

## Discussion

We developed an antibiotic decision-making tool for suspected HAIs, mainly in VLBW neonates. This tool has the potential to significantly reduce antibiotic LOT by providing antibiotic prescription guidance to healthcare workers. The tool incorporates the NeoHoP score and POC CRP stepwise, allowing the physician to make an informed antibiotic decision at the bedside.

In clinical practice, antibiotics are usually started at the time of investigation for suspected HAI, based on clinical suspicion, despite clinical signs being non-specific and inconspicuous. Inappropriate antibiotic use is one of the most important underlying factors driving the worsening global threat of antibiotic resistance [43]. In the ‘Choosing wisely’ survey by Ho et al., it was found that failure to stop antibiotics at 48 h in the absence of proof of infection was one of the top 5 problems contributing to poor antibiotic stewardship [44]. In Sub-Saharan Africa, the scarcity of diagnostic services leads to the frequent prescription of antibiotics based on clinical symptoms rather than confirmed laboratory results, which increases antibiotic use and inadvertently contributes to the rise of antimicrobial resistance [45, 46].

By retrospectively applying the antibiotic decision-making tool to a population of neonates investigated for suspected HAI, we demonstrated a significant theoretical reduction in overall antibiotic LOT by two days. The median observed antibiotic LOT for proven HAI was nine days, shorter than the median of 12 days reported in the NO-MAS-R study, whilst the median LOT for presumed HAI was seven days in both studies [14]. The antibiotic decision-making tool did not affect the antibiotic LOT for proven and presumed HAI. The theoretical reduction in LOT is most pronounced in those neonates where the clinical picture leaves the healthcare worker doubtful about the infection status. In our setting, the decision-making tool can assist healthcare workers in identifying those patients where antibiotic treatment is not indicated and those where antibiotics can safely be stopped after 24 h.

The decision-making tool includes the NeoHoP score, a novel infection prediction score developed as a rule-in screening test for HAI in VLBW [35] and subsequently externally validated in a multicentre study in a more diverse neonatal population [36].

A NeoHoP score of ≥ 2 achieved high specificity and a high positive likelihood ratio, which is well positioned as a ‘rule-in’ test [35]. The score includes using an initial lab CRP, an easily accessible, extensively studied and inexpensive biomarker for infection. However, for this study, we replaced it with a POC CRP test.

The use of CRP in diagnosing HAI remains controversial, mainly because of the slow rise and the emergence of newer and more expensive biomarkers; however, many of these tests are not available in our setting, for example, procalcitonin [47]. Serum CRP concentrations rise within 10–12 h in response to bacterial infections and peak after 36–48 h [48]. Based on this delay in elevation, the ideal time to perform CRP requires careful consideration as serum CRP at the initial investigation of suspected HAI is unlikely to aid diagnosis as a stand-alone test [49]. Performing serial CRP tests after 24–48 h after onset of infection increases the sensitivity and negative predictive value in diagnosing HAI [50, 51]. Procalcitonin serum concentrations increase earlier than CRP, and are produced specifically in response to bacteria, but are not clinically superior to CRP in HAI evaluation [48, 52]. In LMIC settings, however, the cost of PCT tests is prohibitive, and the use thereof may be regulated as a cost-saving measure (unpublished data). Additionally, despite the controversy around the sensitivity of CRP testing, in adult studies it has been shown that CRP testing may have contributed to a reduction in antibiotic prescribing, and is cost-effective provided that the test results are adhered to [53].

Beltempo et al. demonstrated that in VLBW infants investigated for HAI, CRP tests done at presentation and after 24 h, had sensitivity and specificity of 49%, 84%, 76% and 70%, respectively [50]. In our study, the CRP performed better, with sensitivities of 76% and 87.8% at t_0h_ and t_24h_, respectively, and a specificity of 100% for both time points. The improved performance of CRP in our setting may be related to patient selection.

In general, laboratory CRP results can be helpful but are not immediately available, which may affect the utility as an adjunctive test to advise antibiotic prescriptions. In our setting, the time to laboratory CRP test results is affected by multiple pre-laboratory factors, such as the time between the blood draw and the arrival of the sample to the laboratory and the prolonged time between sample registration and analysis. The median time from blood draw to laboratory CRP result availability was more than 6 h, which is longer than the 4–5 h found by Prince et al. [37]. This delay in results increases the likelihood of neonates receiving an additional dose of antibiotic treatment that may have been discontinued had the results been available sooner [37]. Alternatively, POC CRP can be performed at the patient’s bedside, using minimal blood volume and with rapid results. Prince et al. demonstrated that POC CRP may be an alternative to laboratory analysis in a neonatal population [37]. Zecca et al. showed that POC CRP could safely be used as an alternative test in neonates and that gestational age, birth weight, haematocrit, and day of life did not influence the agreement of the results with the laboratory CRP [54]. Serial POC CRP is likely to assist with earlier discontinuation of antibiotic treatment in support of antibiotic stewardship [37]. In our study, the combination of CRP t_0h_ and t_24h_ increased the sensitivity and specificity to 97.4% and 100%, respectively.

By combining the NeoHoP score and CRP t_24h_ in this decision-making tool, we achieved a sensitivity of 98.7% and a specificity of 97.8%. Although serial CRPs had a higher specificity than the decision-making tool, using CRP tests in isolation cannot be recommended when making antibiotic decisions as they cannot safely be used in the decision to not start antibiotics due to the high false negative rate of CRP t_0h_. With the decision-making tool, 40.7% of neonates’ antibiotics could theoretically safely be withheld, reducing the need for invasive procedures such as sterile blood collection for cultures and indwelling intravenous catheters.

A strength of our study is the inclusion of in-patient neonates with presumed and proven HAI with a high risk of morbidity and mortality. The inclusion of presumed HAI is aligned with the practice in many LMICs, where many healthcare providers rely on auxiliary tests such as CRP to guide their antibiotic decision-making. In our unit, the presumed HAI is equally as prevalent as the proven HAI [55]. Notably, urinalysis and chest X-rays were performed based on the clinical presentation of the neonate, and the results are not immediately available in our setting. Based on this knowledge, these investigations were not included in the development of the score and the subsequent validation.

A study limitation is the theoretical evaluation of the antibiotic decision-making tool to determine its possible impact on antibiotic LOT. Still, our findings suggest that the tool may benefit substantially if used in real time. Neonatal healthcare providers should understand that decision-making tools are merely a clinical support aid, not a substitute for clinical understanding or experience. Where healthcare providers are uncertain and commence antibiotic therapy empirically, the tool could still be used at 24 h as a second screening time point to evaluate for the possible early cessation of antibiotic therapy.

Additionally, this was a single-centre study performed at a tertiary institution in a low-resource setting, which may limit the generalizability of our study’s findings to other settings.

## Conclusions

We developed an antibiotic decision-making tool for suspected HAI in neonates that has the potential to reduce antibiotic LOT in neonates significantly. This tool may be necessary for healthcare providers working in low-resource settings to minimise the unnecessary duration of antibiotics and is well-positioned to improve antibiotic stewardship. For this reason, low-cost POC CRP should be considered a priority intervention in LMIC neonatal units, especially where laboratory CRP is unavailable.

## Electronic Supplementary Material

Below is the link to the electronic supplementary material.

Supplementary Material 1: Table 1. Description of positive blood cultures and microorganisms (*n* = 22). Table 2. Theoretical impact of the antibiotic decision-making tool on antibiotic LOT in neonates with suspected healthcare-associated infection, compared to observed antibiotic LOT.

Supplementary Material 2: Fig. S1. Decision tree predicting the presence of healthcare-associated infection in neonates. *HAI indicates healthcare-associated infection; NeoHoP score*,* Neonatal Healthcare-associated infection Prediction score; t*_*0*_, *time of presentation; POC CRP*,*point of care C-reactive protein.*

## Data Availability

Data availability statement: The original data presented in this study are available upon reasonable request from the corresponding author. Data will be anonymised.
