# Supplementary material for: Theoretical impact of a bedside decision-making tool on antibiotic use for suspected neonatal healthcare-associated infection: an observational study
Source: BMC Pediatr. 2025 Jan 21;25:52. doi: 10.1186/s12887-024-05323-8 (PMC11749325; doi:10.1186/s12887-024-05323-8)
Supplement: Supplementary file 1 — Supplementary Material 1: Table 1. Description of positive blood cultures and microorganisms (n = 22). Table 2. Theoretical impact of the antibiotic decision-making tool on antibiotic LOT in neonates with suspected healthcare-associated infection, compared to observed antibiotic LOT. [file 12887_2024_5323_MOESM1_ESM.pdf]

Supplementary table 1. Description of positive blood cultures and microorganisms (n=22)

**Organism**	**Number (%)**
**Gram-negative organisms**	6 (27.3)
Klebsiella spp.	5 (22.7)
Escherichia coli	1 (4.5)
**Gram-positive organisms**	12 (54.5)
Methicillin-susceptible *Staphylococcus aureus*	2 (9.1)
Methicillin-resistant *Staphylococcus aureus*	1 (4.5)
CoNS	8 (36.4)
Enterococcus faecalis	1 (4.5)
Polymicrobial, n (%)	*4 (18.2)*

CoNS indicates coagulase-negative staphylococci

Supplementary Table 2: Theoretical impact of the antibiotic decision-making tool on antibiotic LOT in neonates with suspected healthcare-associated infection, compared to observed antibiotic LOT

	Observed antibiotic LOT (days), median (IQR)	Theoretical antibiotic LOT (days), median (IQR)	p-value
**Total**	3 (3-7)	1 (0-5)	<0.001
**No HAI**	3 (2.5-3)	0 (0-1)	<0.001
**Any HAI**	7 (5-10)	7 (5-10)	1.000
Proven HAI	9 (9-14)	8 (9-14)	1.000
Presumed HAI	7 (5-9)	7 (5-9)	1.000

LOT indicates length of treatment; IQR, interquartile range; HAI, healthcare-associated infection
