# Supplementary material for: Theoretical impact of a bedside decision-making tool on antibiotic use for suspected neonatal healthcare-associated infection: an observational study
Source: BMC Pediatr. 2025 Jan 21;25:52. doi: 10.1186/s12887-024-05323-8 (PMC11749325; doi:10.1186/s12887-024-05323-8)
Supplement: Supplementary file 2 — Supplementary Material 2: Fig. S1. Decision tree predicting the presence of healthcare-associated infection in neonates. HAI indicates healthcare-associated infection; NeoHoP score, Neonatal Healthcare-associated infection Prediction score; t0, time of presentation; POC CRP,point of care C-reactive protein. [file 12887_2024_5323_MOESM2_ESM.docx]

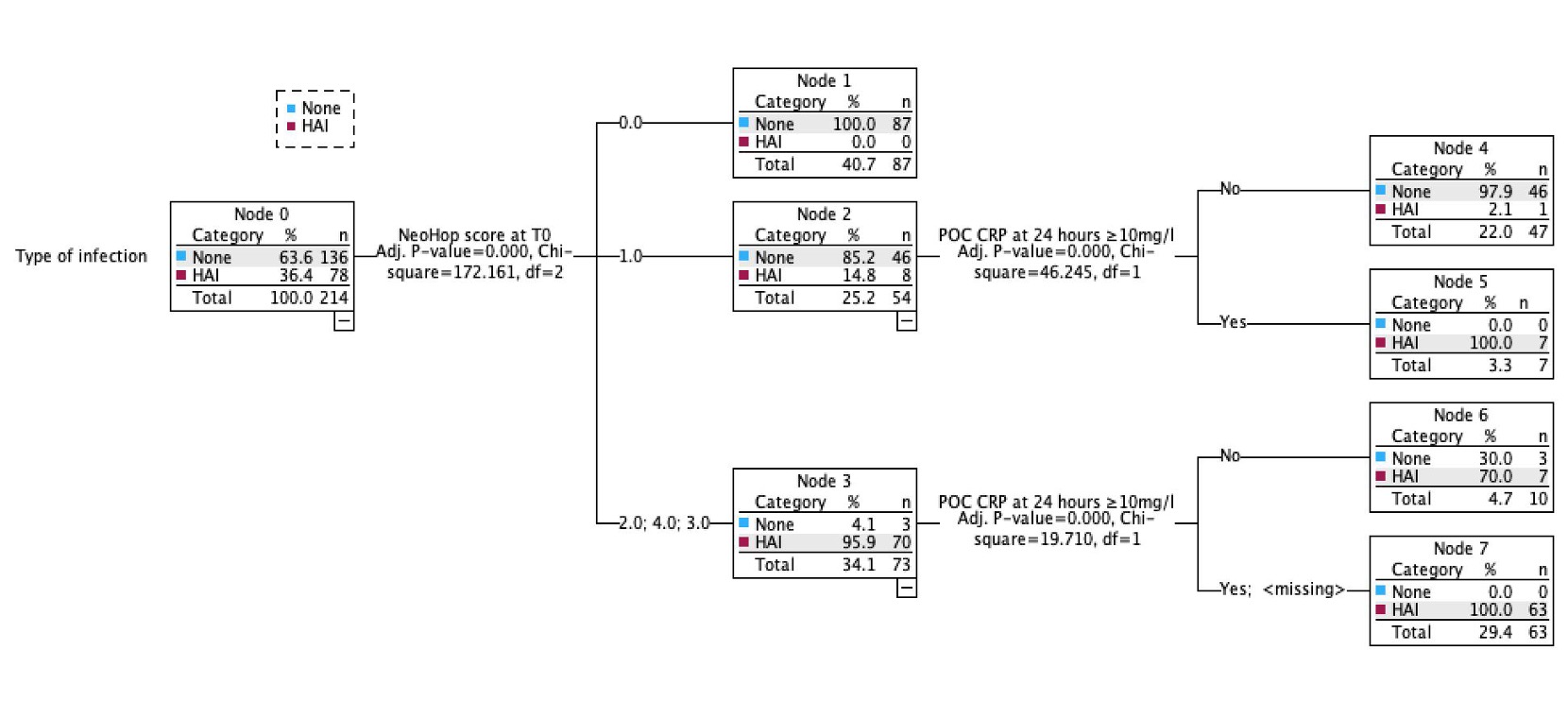


**Figure S1: Decision tree predicting the presence of healthcare-associated infection in neonates.**

*HAI indicates healthcare-associated infection; NeoHoP score, Neonatal Healthcare-associated infection Prediction score; t_0_, time of presentation; POC CRP, point of care C-reactive protein*
